# Recognition of a Person Wearing Sport Shoes or High Heels through Gait Using Two Types of Sensors

**DOI:** 10.3390/s18051639

**Published:** 2018-05-21

**Authors:** Marcin Derlatka, Mariusz Bogdan

**Affiliations:** 1 Department of Biocybernetics and Biomedical Engineering of the Faculty of Mechanical Engineering at Bialystok University of Technology, 15-351 Bialystok, Poland; 2 Department of Automatic Control and Robotics of the Faculty of Mechanical Engineering at Bialystok University of Technology, 15-351 Bialystok, Poland; m.bogdan@pb.edu.pl

**Keywords:** biometrics, human gait recognition, ground reaction forces, Microsoft Kinect, high heels, fusion data, ensemble classifiers

## Abstract

Biometrics is currently an area that is both very interesting as well as rapidly growing. Among various types of biometrics the human gait recognition seems to be one of the most intriguing. However, one of the greatest problems within this field of biometrics is the change in gait caused by footwear. A change of shoes results in a significant lowering of accuracy in recognition of people. The following work presents a method which uses data gathered by two sensors: force plates and Microsoft Kinect v2 to reduce this problem. Microsoft Kinect is utilized to measure the body height of a person which allows the reduction of the set of recognized people only to those whose height is similar to that which has been measured. The entire process is preceded by identifying the type of footwear which the person is wearing. The research was conducted on data obtained from 99 people (more than 3400 strides) and the proposed method allowed us to reach a Correct Classification Rate (CCR) greater than 88% which, in comparison to earlier methods reaching CCR’s of <80%, is a significant improvement. The work presents advantages as well as limitations of the proposed method.

## 1. Introduction

In the world of constantly developing technology biometrics occupies a special place. Biometrics understood as the recognition of a particular person is already in use in forensic [1,2] as well as commercially (by ATMs, for example). Among the various field of biometrics the human gait is especially intriguing [3,4]. It is the result of a coordinated cooperation between the nervous and the musculoskeletal systems and it is accepted that after maturity all the way to advanced age it generally remains unchanged. As early as the 1970s research had shown that the way a person moves is to a great degree individual and allows the identification of a person [5]. A number of works dealing with the subject of identifying people in relation to the way they move have been published since that time [6,7,8,9,10]. Connor and Ross categorized these studies on the basis of sensors used to obtain measurements and divided them into methods using [11]:

- video cameras [12,13],
- the measurement of pressure exerted by a person’s foot on the ground [14,15,16],
- accelerometers and other wearable devices [17,18,19],
- audio [20,21].

Works connected with the biometrics of the human gait mainly concentrate on creating systems guaranteeing the highest accuracy possible. Of course, the methodology which allows this relies directly on the character of the registered data. In case of signals recorded using video sensors the Gait Energy Image (GEI) representation has been successfully employed. GEI is obtained through a simple average of silhouettes during walking. Modifications of this method which improve GEI effectiveness [22] are also utilized. Wavelet transform [23], fuzzy logics [24] or dynamic time warping (DTW) [25,26] are some methods which are used to preprocess measured time series.

When it comes to classifiers hidden Markov models (HMM) [27], support vectors machine (SVM) [28], k-nearest neighbors [29,30], neural networks [31] or deep learning [32] are often utilized. Additionally, to improve the quality of obtained results, ensemble classifiers are being used more and more often [33,34]. These are systems which consist of several homogeneous or heterogeneous classifiers used for the realization of the same classification task. A decision of a set of classifiers is made on the basis of decisions reached by individual classifiers, for example, on the basis of the majority vote.

The most frequent sets of classifiers seen in biometrics are those which, to identify a person, simultaneously use various types of biometrics. Most often encountered works combine two or more varying biometrics. The recognition of people on the basis of face and palm print [35], face and gait [36] or shape of hand and palm print [37] can be seen as examples of bimodal biometrics. The use of multi-biometrics can be found in the work of [38]. It is also possible to encounter biometric systems utilizing a single human feature in which the input to classifiers is obtained through bagging [39] or boosting [40]. The measurement of that same phenomenon is less often gained through various sensors. To recognize gait in their work Hoffman et al. [21] used visual RGB image sequence, depth image sequence and four channel audios. In [41] to human gait recognition the GRFs and some anthropometric features obtained from Kinect have been used. The obtained results showed that in the majority of examined scenarios combining information from sensors varying in physical character improved recognition results.

Regardless which measuring methods are used to preprocess data or classifiers the quality of biometric systems based on the way a person moves is still greatly influenced by the footwear the subject is wearing. From biomechanical point of view the greatest change becomes visible in regard to the movement of women wearing high-heeled shoes. According to [42] an increase in the height of the heel in a woman’s shoe causes a decrease in her walking speed and the length of her stride while keeping nearly an identical cadence. In [43] it has been noticed that the increase in the height of the heel causes an increase of extreme values of all components of ground reaction force. Additionally, Barton et al. [44] showed that heel lifts exerted greater muscle activity before and after the heel strike. Significant rise in the activity range of muscles of the lower limbs was also observed in [45]. Of course, the way a person moves walking in high-heeled shoes is also influenced by that person’s experience. In [46] it has been shown that a change of footwear has a greater impact on the way a person walks if the subject is less experienced. Similarly, de Oliveira et al. [47] recoded the influence of high-heeled shoes on lumbar lordosis and pelvis position dependent on how often such footwear was worn. In case of experienced users hyperlordosis and pelvic anteversion was noted while in inexperienced users rectification of the lumbar spine and pelvic retroversion was reported. It must also be mentioned that in Simonssen’s et al. work no significant difference in the electromyographic activity of muscles (EMG) or joint movements between experienced and inexperienced high heels users has been recorded [45].

When it comes to biometrics problems connected with the impact of footwear change on the accuracy of identifying people is not often brought up. Using an RGB camera Sakar et al. [48] studied a group of 122 people mainly men of which slightly more than half walked in two different types of shoes (sneakers, sandals, high heels, etc.). Unfortunately, during the experiment various people could walk in different types of footwear, therefore, the conclusion of the article stating that a change in footwear has little impact on the accuracy of identifying people is of limited value. Bouchrika and Nixon [49] noticed that the influence of footwear on the correct recognition of a person depends on its type. Although their study was performed on a group consisting of only 20 people (440 video sequences) their results showed that Correct Classification Rate (CCR) falls from 83.33% in trainer shoes to only 46% in flip flops. Gafurov et al. [50] utilized data from accelerometers to identify a group of 30 men with each of them walking in four different types of shoes. In case of limiting data to a particular type of footwear Equal Error Rate (EER) was from 1.6 to 6.1%. However, inclusion of all types of shoes caused a significant decrease in the system’s accuracy and EER increased in range from 16.4 to 23.6%. Connor conducted barefoot gait recognition and shod-foot recognition when the shoe used in training was the same or different from the test shoe. In the first instance EER was 2.1% (15 people) and in the second it ranged from 11.4 to 15.9% (13 people) with a study group consisting mainly of men.

Studies in which high-heeled shoes are considered are even rarer. In his work Kim [51] used a motion capture system (Vicon, Oxford, UK) to identify people from a group of 10 (160 gait strides) who walked in four types of shoes having various heel heights. The results obtained for the greatest difference in heel height allowed identification in only 72.5% of cases. Cronin [42] conducted a study on a group of 125 people on the basis of data obtained from a video camera. This research concerned, among others, the impact of the type of footwear on the accuracy of a system for the identification of people. Types of shoes taken into account in the study included: normal shoes, formal shoes (high-heeled shoes for females and dress shoes for males) and casual wear (slippers). CCR for those individual shoe types was respectively: 81.25%, 78.84% and 80.65%. In [46] the ground reaction force and ensemble classifiers have been used to identify people with consideration for three research scenarios. The first one examined only gait in sport shoes, the second assumed that the learning set contains data describing gait only in sport shoes and the testing set also includes data from movement in high heels, while the third permitted both types of footwear in both sequences. The percentage of accurate recognition was respectively 98.87%, 69.21% and 98.96%.

A review of literature shows that there is a significant gap in works connected to human gait recognition related to the recognition of the gait of women walking in high-heeled shoes. This became our motivation for this paper to project a biometric system which will, with high accuracy, identify women walking in high-heeled footwear on the basis of data gathered through two sensors: force plates and Microsoft Kinect. The additionally presented biometric system has been validated through a secondary study performed on a selected sub-group of subjects.

## 2. Basics of Human Gait

Typical gait of people is distinctive in the coordinated, repeatable movement of the trunk and limbs used to move the body, maintain it in a vertical position with the least possible expansion of energy. While walking the lower limbs function as supports and a means of propulsion. They work in an alternating manner and their movements are cyclical which means that the same movements are performed in particular time increments. From the biomechanical point of view the human gait is perceived as a spatial, cyclical motion act in which the center of gravity of the torso is momentarily shifted beyond the support plane of the lower limbs to, within the next stage, regain balance along with performance of forward movements in the direction of stepping. The forward progression of the body begins at the moment when the bearing foot leaves the ground with the simultaneous raising of the heel and the shifting up of the entire body’s center of gravity. At the same time, the second, unburdened limb swings forward until its heel touches the ground. In effect there is the lowering of the foot with the simultaneous shifting of body mass. During the performance of these alternating movements the trailing leg becomes the leading leg and vice versa.

Within the biomechanical gait analysis it has been accepted that the walking cycle is measured from the moment the heel of one lower limb touches the ground (in respect to physiological gait) to the moment until it touches the ground again. During this time both limbs go through the support phase and the swing phase in which the limb is shifted above the ground. The support phase lasts approximately 60% of the entire cycle and can be broken down into the following sub-phases:

- The Initial Contact (IC)—in this phase the foot comes into contact with the ground. In a typical gait the initial contact is made with the heel which is the reason this phase is often also called the Heel Strike (HS).
- The Loading Response (LR)—the foot is rotated forward to maintain the speed of the body’s forward momentum and cause its full contact with the ground. LR lasts from IC until the moment the toes of the other foot lose contact with the ground. This sub-phase completes the double-support phase where both legs touch the ground. LR lasts from 0 to approximately 10% of the entire cycle.
- The Midstance (MSt)—begins the single support phase. It is the phase lasting from the time the toes of the opposite foot lose contact with the ground and the moment when the body weight is aligned over the forefoot. The analyzed foot lies flat on the ground. It is the period from about 10 to 30% of the entire time of the gait cycle.
- The Terminal stance (TSt)—starts with the heel-off, followed by the limb bending forward and the trail leg (opposite leg) becomes the leading leg. The phase ends with the initial contact of the opposite leg. TSt lasts from 30 to approximately 50% of the gait cycle.
- The Preswing (PSw)—begins with IC of the opposite leg and finishes with the toe off of the analyzed lower limb. Interval: 50–60% of gait cycle.

The transfer phase lasts about 40% of the entire gait cycle and can be divided into the following sub-phases:

- The Initial swing (ISw)—begins with the lifting of the foot off the ground. Thanks to the bending of the limb in the knee and the hip the foot is shifted forward. This phase ends when the swinging foot is opposite the stance limb. It is assumed that this phase lasts from 60 to 73% of the gait cycle.
- The Mid swing (MSw)—This phase begins when the swing foot is opposite the stance leg and ends when the moving limb is forward and the tibia is vertical. This phase lasts from 73 to 87% of the gait cycle.
- The Terminal swing (TSw)—is the last phase of the swing which ends with the initial contact of the leg being analyzed. This phase lasts from 87 to 100% of the gait cycle.

During walking it is possible to see a change in the distance between the top of the person’s head and the ground. The maximum distance is measured during the midstance and the minimum distance occurs during the double-support phase. According to [52] the difference between those two distances can be as much as 9.5 cm.

## 3. Materials and Method

### 3.1. Sensors and Measured Data

#### 3.1.1. Force Plate

The force generated during walking between the foot and the ground is called the ground reaction force or GRF. To measure this force plates made by the Kistler Company (Winterthur, Switzerland) utilize four piezoelectric sensors located in the corners of the platform. The signal measured by the sensors is employed to represent three components of GRF: anterior-posterior Fx, vertical Fy and lateral Fz.

**Figure 1:**
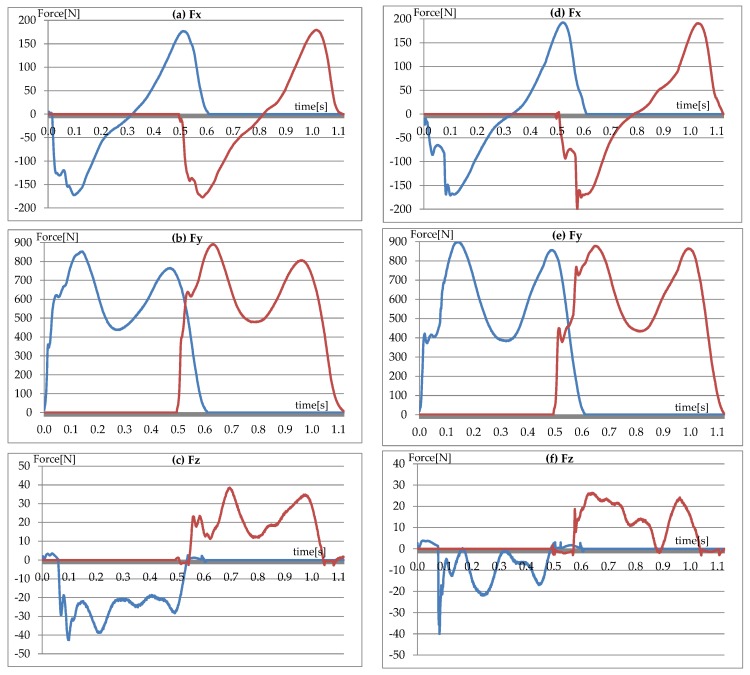
Components of GRF in: (**a**,**d**) anterior/posterior; (**b**,**e**) vertical; (**c**,**f**) medial/lateral direction of the left lower limb (blue line) and of the right one (red line) in sport shoes (**a**–**c**) and high heels (**d**–**f**). Data derived from the same subject.

Maximum values for the vertical component Fy correspond to the moments of: transferring the entire body weight onto the analyzed limb (first maximum—maximum of the overload phase) and the load of the forefoot (the heel is not in contact with the ground) right before the toes off (the second maximum—maximum of propulsion). In a typical gait these maximum values reach approximately 120% of body weight. This is the result of the dynamics of the phenomenon and the need of maintaining balance while walking. Hence the value of the reaction forces is greater than the force of gravity (weight). Half way through the supporting phase the entire active surface of the foot is in contact with the ground. This is a period of unloading (minimum of the unloading phase) and the decrease in the force value to below 100% can be seen on the Figure 1 The anterior-posterior Fx component consists of two phases. During the first its value is negative when it is opposite to the direction of movement. It is the result of the deceleration of the analyzed lower limb. The minimum of the deceleration phase is most often reached right before the occurrence of the maximum of the overloading phase for the vertical Fy component. Similarly, during the second phase the anterior-posterior component shows positive values. It is then that the process of acceleration begins concluded by pushing off the ground with the toes. During this entire interval the turn of the Fx force corresponds to the direction of movement. The maximum of the acceleration phase occurs in the initial phase of push the toe offs. This happens right after the maximum of propulsion for the vertical Fy component. The value of the Fx component is equal to zero at the moment when the analyzed limb passes the trail leg. This more or less corresponds to the moment of the minimum of the unloading phase for the vertical Fy component. Extreme values of the Fx component reach approximately 20% of the weight of the test subject.

The value of the lateral Fz component depends on the limb being analyzed. Assuming that movement occurs in the direction determined by the orientation of the Fx force than the values of the Fz component will be positive for the left leg and negative for the right leg. The exceptions include the moment of initial contact and the moment when the toes leave the ground where the foot is slightly supinated. The value of the Fz force depends on the manner in which the test subject places his feet. This force should be greater both in the event of pronation as well as the abduction of the foot. Extremes for Fz use the same nomenclature as those for the vertical Fy component; maximum of the overloading phase, minimum of the unloading phase and maximum of the propulsion phase. The values of these forces are about 10% of the body weight of the test subject.

Measurements made as part of this study were performed using two Kistler platforms with the dimensions of 60 cm × 40 cm registering data with a frequency of 960 Hz.

#### 3.1.2. Microsoft Kinect v2

**Figure 2:**
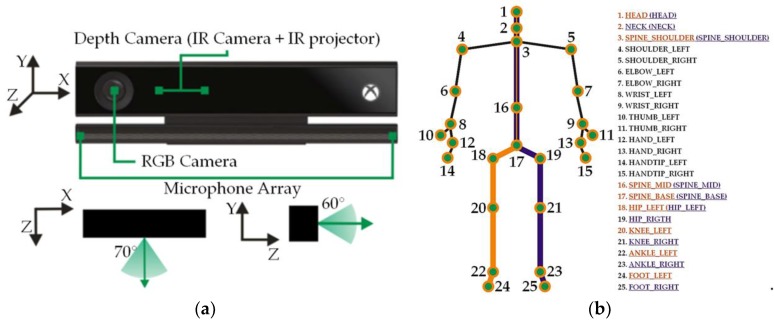
Microsoft Kinect v2: (**a**) Kinect structure and visual field marking; (**b**) the location of 25 parts of the body in Kinect v2.

Kinect from Microsoft (Redmond, Wa, USA) in the v2 version (Xbox One) is the successor of Kinect v1 (Xbox 360). Due to the price and opportunities offered (sensor set: RGB camera, depths, directional microphones—Figure 2a), similarly to the previous version, it is very popular: It has found a wide application in various types of applications related to, among others, object recognition and reconstruction, 3D reconstruction and many others [53,54,55]. In the case of human recognition based on gait, it significantly expanded the approach area in methods based on a model description (model-based approaches) [56,57,58,59]. This is related to the ease of obtaining information about depth and skeletal data without the need for implementation computationally complex processing algorithms and video analysis algorithms. Kinect v2 sensor allows tracking and construction of virtual 3D skeleton in real time (Figure 2b). In 2014, Microsoft released the Kinect for Windows SDK 2.0 version. The SDK software [60] contains the NUI Skeleton library, which allows obtaining information about the location of the 25 parts of the body (joints) relative to the sensor (Figure 2b).

In Table 1, the Kinect v2 features relevant from the point of view of the performed measurements are listed. In general, the individual Kinect v2 parameters and thus the skeleton tracking accuracy has been improved in relation to the previous generation of the sensor. In addition, the ability to register the number of skeletal joints has been increased by 5.

Along with the improvement of individual sensor parameters, the method of depth measurement has a great influence on the quality of the skeleton tracking as well. In Kinect v2, unlike in Kinect v1 (the technology used is based on structured lighting, pattern deflection and triangulation), Time of Flight technology—ToF (ToF camera) is used. The ToF system is based on the measurement of the return time of the infrared electromagnetic radiation beam reflected from the illuminated object. Thanks to these combined treatments (improvement of parameters + new method), the quality of skeleton tracking has been improved in relation to Kinect v1 [61,62] (lower image degradation due to lighting effect, higher quality and accuracy of depth image, reduction by ¼ of blur caused by motion and much larger field of view).

For the needs of the research, an (C#) application was created. The application is based on the official 2.0 Microsoft SDKs (Software Development Kits, freely available for Kinect v2) and it allows the following activities:

- simultaneous capture of image data stream from the RGB camera and depth camera of the Kinect controller;
- skeletal tracking;
- the choice of image resolution from RGB camera and a depth camera;
- a description of the figure movement—calculating and displaying the registered figure;
- displaying graphs from earlier collected data;
- recording specific (significant) parameters and status of tracked points (joints) to an Excel file (.xlsx or .cs extension) or notepad (.txt extension), including registration time (DateAndTime::Now, .Net Framework).

**Figure 3:**
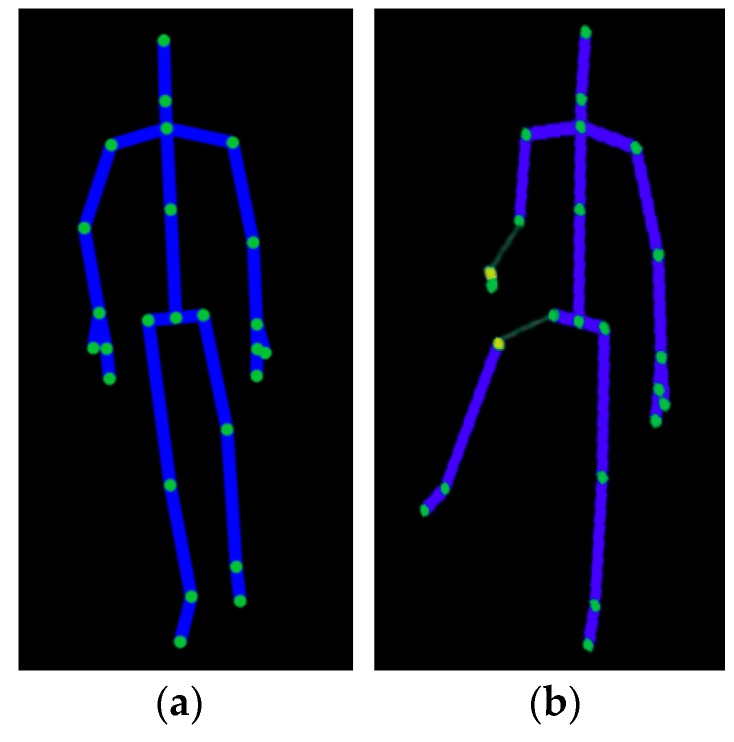
Preview of the user’s skeleton when: (**a**) all joints are properly tracked; (**b**) Kinect is not able to determine the position of certain joints.

For the purposes of this article, it was decided to choose only the body height (selected anthropometric characteristic). It should be noted that static data are fixed i.e., it is not dependent on the type of human gait (it is often of non-constant speed and non-constant frequency) and on its characteristics (speed of locomotion, stride length, etc.). In the course of the research, it was found that, unlike Kinects v1, Kinects v2 do not interfere with each other, which makes it possible to freely adjust them in relation to each other. In addition, the application gave a preview of the entire skeleton. “Bones” can take two colours: blue—for those correctly detected and yellow—when the sensor is not able to accurately determine the position of a joint (Figure 3).

The .txt file saved all information related to the tracking of the person, including skeleton joint tracking states (fully tracked, inferred, or not tracked). Skeleton joint tracking was used in offline processing. To determine the length of individual body parts, only joints (Figure 2b additionally denotes sections that were taken into account when determining the body height—dark purple and orange lines) classified as fully tracked were taken into account. Therefore, in order to be able to determine, for example, the length of the right lower limb as fully tracked, there had to be joints marked: 19 (hip right) and 21 (knee right) (see Figure 2b).

**Figure 4:**
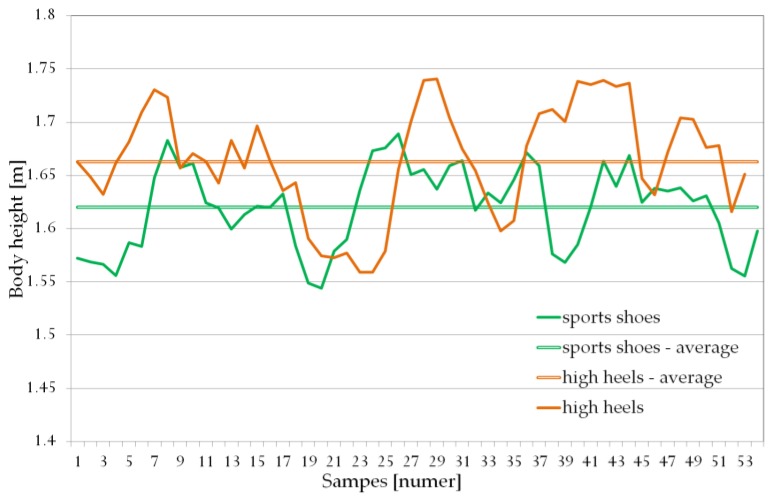
Changes in body height during walking.

For the lower limbs, especially in areas deviating from the optical axis (in the areas at the border of the sensor’s field of view) during the movement, the need to use information from both sensors was emphasized. Individual points enabling the determination of sections of the lower limbs were determined based on their correct detection (skeleton joint tracking states: fully tracked). In the case of detection errors of the body part (or body parts) of one of the lower limb, to calculate the body height, the correctly determined body part was taken from the correspondent part of the other leg and from the values determined by the second Kinect. If the above conditions were not met, the algorithm was to omit this measurement. However, in the conducted studies, such a case did not occur. In a situation in which both Kinect sensors correctly detected individual body parts, the average value for the given body part was determined. Due to the bandwidth required by Kinect v2, each sensor was connected to a separate computer with identical technical specifications (Windows 10 OS, Intel Core i7-4700MQ, 16 GB RAM, Kinect SDK 2.0). The application was simultaneously run by one user on two computers using two computer mice with shortcut connection. It required a relatively simple interference in the construction of a computer mouse. It was about detecting the left mouse button pressed (then the contacts are shorted and the current flows in the system) and sending the pulse with the cable to the second mouse (that is passing the current despite the fact that there was no short-circuit), which corresponded to almost simultaneous pressing the left mouse button on the second mouse same time. The delay caused by the propagation time by the cable connecting the two mice in comparison to the operating frequency of Kinect v2 was not significant. Almost simultaneous starting of the applications allows to treat both measurements as synchronized in time. Because during one experiment Kinect registered more than one step, even possible time shifts in the time course would have a much smaller impact on the average body height than the type of shoes in which the measured person was moving. It is also worth noting that the registration of the time of registration (DateAndTime::Now, .Net Framework) enabled full control over the offline synchronization of measured data (measurements). The measurement results of body height of people while walking in sport shoes and high-heeled shoes have been presented in Figure 4.

Figure 4 shows the dynamic change in body height during walking (the relationship expressed in meters). This change is caused by, among others, the previously mentioned natural change in human body height during the gait cycle. In addition, the entire measurement is burdened with quite a big error, which in selected moments reaches a value of a few centimeters. However, it should be emphasized that this error does not significantly affect the results obtained. The location of Kinects during the measurement makes it possible to register more than one gait cycle so that the received average values are close to the actual ones. The average body height value determined with two Kinects, in the case of walking in sports footwear, was 162.1 cm (actual measured body height 160.9 cm). However, in the case of the same person walking in high heels, the average body height value was 166.1 cm with the actual measured height of 166.7 cm.

**Figure 5:**
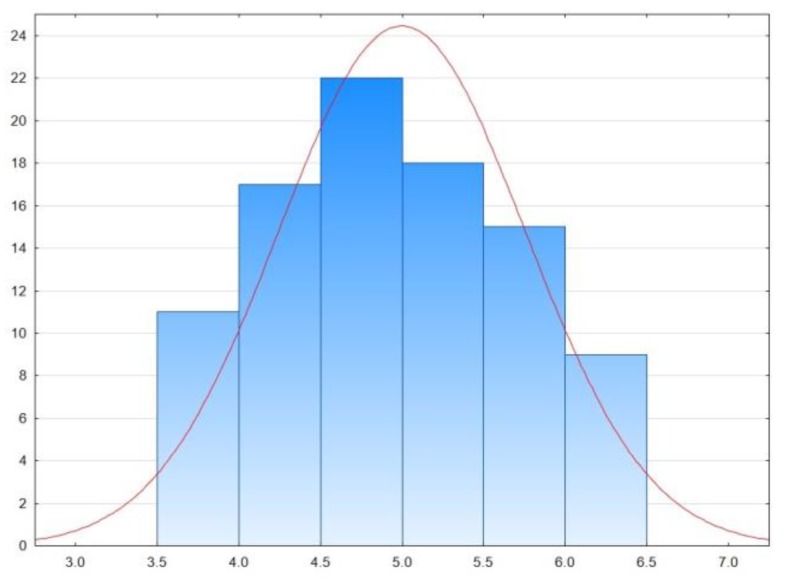
Histogram of the difference in the body height of people walking in sport shoes and high-heeled footwear with a height of 8–10 cm; average value = 4.988 cm; σ = 0.7504 cm.

The difference in the average value of body height of people walking in sport shoes and high-heeled footwear has been presented on the graph below (Figure 5). The Shapiro-Wilk test showed that the presented data exhibits normal distribution. Statistical analysis was performed using Statistics 13.5, and the statistical significance was set at *p* < 0.05.

The average difference in the body height of a person walking in high heels with a heel height of 8–10 cm and in sport shoes was less than 5 cm. This difference is not equal to the heel height which is caused both by the thickness of the sport shoes’ soles as well as the inaccuracy of measurements made using Microsoft Kinect v2. It should be said that that the desired result of the proposed method is not the actual height of the person being measured but rather the ability to differentiate between individuals and dependence on the type of the footwear which the person is wearing. The most important is the fact that assumed range of differences of ±3σ will allow consideration of all cases occurring in the data set.

### 3.2. Data Processing

Ground reaction forces registered using force plates made by the Kistler Company are in the form of time series: x_1_, x_2_, …, x_n_, where n is the number of samples. Generally, the duration of the supporting phase for various steps differs which is the reason that the representation of the gait cycle consists of time series of varying lengths. Therefore, to determine GRF similarities of various gait cycles a well-known algorithm of dynamic time warping (DTW) was used. DTW calculates an optimal warping path which allows the transformation of one time series (the one being analyzed) into a different one (referential). The cost of such transformation is smaller if the two time series being compared are similar. Hence the cost of imitation has been utilized as the measure of distance.

Within this work fragments concerning phases were chosen from obtained GRF’s: Mid stance and Terminal stance separately for each leg. The duration of individual phases has been presumed in accordance to the values presented in Section 2. We assume that *ρ_v,s_* signifies a distance between two time series describing the GRF in the *v* phase of the gait cycle for the *s* limb. This distance has been calculated using the following formula:

(1) $$\rho_{v,s\;=\;\sum_{m=1}^{M}{\mathrm{DTW}}_{m}}$$

where DTW_m_ is the distance between two time series calculated for the *m* component of GRF. *M* is equal to the number of considered components. In this work we made use of all components therefore *M* = 3.

Additionally, the distance of the entire stride without dividing it into individual phases or limbs has also been determined (in that case *M* = 6 in Equation (1)). This resulted in 5 distances: *ρ_MSt,L_*; *ρ_TSt,L_*; *ρ_MSt,R_*; *ρ_TSt,R_*; *ρ_Stride_*.

### 3.3. Data Fusion

Measurements made using devices described above can be presented as a six element vector:

V = [*ρ_MSt,L_*; *ρ_TSt,L_*; *ρ_MSt,R_*; *ρ_TSt,R_*; *ρ_Stride_*; *BH*] (2)

where *ρ_MSt,L_* is the distance between two time series calculated for the left lower limb during Mid Stance phase; *ρ_MSt,R_*—the distance between two time series calculated for the right lower limb during Mid Stance phase; *ρ_TSt,L_*—the distance between two time series calculated for the left lower limb during Terminal Stance, *ρ_TSt,R_*—the distance between two time series calculated for the right lower limb during Terminal Stance, *ρ_Stride_*—the distance calculated for both legs without division into phases, *BH*—subject’s body height.

The data is in the form of individual values hence there was no need to synchronize measurements between those obtained from the force plates and those from the Microsoft Kinect devices. The method of identifying people proposed by this work is carried out in two stages and utilizes data from sensors mentioned above. Within the first phase there is the recognition of the type of footwear which the test subject is wearing. Then, as part of the second phase, through the consideration of data from vector *V* the actual identification process occurs.

Identification of footwear was done using the vertical and the anterior-posterior components of GRF of both legs generated during the LR phase of the gait cycle. The decision was made after an analysis of time series’ values of that phase. To develop the input vector for the classifier the coefficients of a polynomial of 5th degree that fits the F_c,s_ = f(time) best in a least-squares sense: [a_c,s,5_; a_c,s,4_; a_c,s,3_; a_c,s,2_; a_c,s,1_; a_c,s,0_] where c—designates a component of GRF, c ∈ {x,y} and s—defines the limb s ∈ {L,R} were utilized [63]. The choice of the polynomial to the 5th degree was dictated, on the one hand by the accuracy of representing the time series and, on the other, by the possibility of overfitting the classifier in the event of the input space being too large. As a result an input vector consisting of 24 elements was obtained. 10-fold cross-validation was used to bulid the classifier where the registered inputs from the same person were always within the same set.

The aforementioned second phase of identification started from the results of footwear recognition of the test subject. In the event where a classifier determined that the person was walking in high heels than a correction of that person’s height was made. On the basis of the data presented in Figure 4 the average value of the difference in the height of a person walking in sports shoes or in high-heeled shoes is 4.988 cm (σ = 0.7504 cm). Since this is a certain approximation of a phenomenon the rounded up value of 5 cm and the acceptable deviation of ±2 cm (which is a value only slightly lower than ±3σ) were used in subsequent calculations:

(3) $$BH_{norm}=\{\begin{array}{ll} BH_{measured} & \;\mathrm{if}\;y=0\\ BH_{measured}\;-5 & \;\mathrm{if}\;y=1 \end{array}$$

where *BH_norm_* is the height after modification; *BH_measured_* is the person’s height measured using the Microsoft Kinect v2 device; *y* is the value of classifier output (*y* = 1 for high-heeled footwear and *y* = 0 for sport shoes).

**Figure 6:**
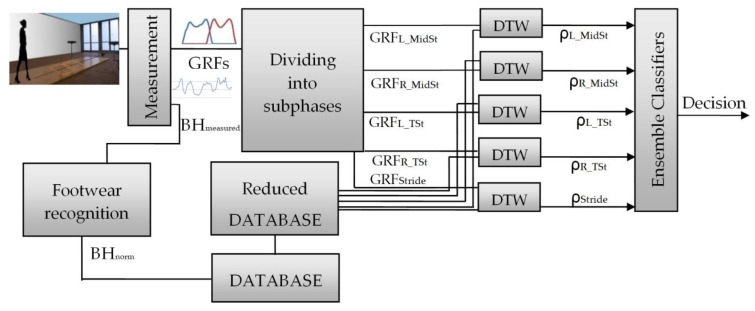
The scheme of the experiment.

The resulting *BH_norm_* was used to limit the number of potential recognized people present in the data base through not taking into consideration for the final solution those women whose body height differed by more than ±2 cm. Hence all subsequent calculations were performed on a ‘Reduced Database’. The scheme of the experiment is presented in Figure 6.

### 3.4. Human Recognition

The recognition of people comes down to the issue of classification where the number of classes is equal to the number of people present in the database (people who, for example, have access to resources). Since DTW allows the designation of the distance between two time series it is natural to use a classifier like *k*-Nearest Neighbor (kNN). On the basis of the affiliation of its nearest neighbors to the *k* classes kNN makes a decision about assigning the considered subject to one of the classes.

Since after preprocessing we obtained 5 distances it only seemed natural to utilize an ensemble of classifiers which consisted of 5 k-NN classifiers. *K* labels defining the affiliation to nearest classes of ‘points’ within a state space are delivered to the inputs of every database classifier. The decision of the entire set of classifiers was made on the basis of a weighted vote (weights based on rank order). The weighted value connected to every label depended on rank *R* in a particular base classifier. The final decision was the class label with the largest total of weights:

(4) $$cl=\arg\max\left(\overset{5}{\underset{j=1}{\sum }}w_{j}\cdot d_{j,i}\right)$$

where *cl*—class label; *k*—the number of neighbors, *w_j_* = [*w*_1_, …, *w_R_*, …, *w_k_*]—weights, which are calculated from the following formula:

(5) $$w_{R}=\frac{k+1-R}{k}$$

where *R*—indicates the rank for *j*-th classifier, *R* = {1, 2, …, *k*}. *d_j,i_*—decision of the *j-*th classifier, which indicates the *k* nearest neighbors, *d_j,i_* ∈ {0,1}. If *j-*th classifier chooses class *i* then *d_j,i_* = 1 otherwise *d_j,i_* = 0.

It was accepted that a person is unrecognized (which meant that the person was not in the database) if at least two classes had the same total weight or if the final total was smaller than the arbitrarily chosen threshold *Th*. In those cases the person was given a ‘NONE’ label. The accepted threshold permits a minimum required level of similarity to consider the person being scrutinized as identified.

### 3.5. The Study Group

**Figure 7:**
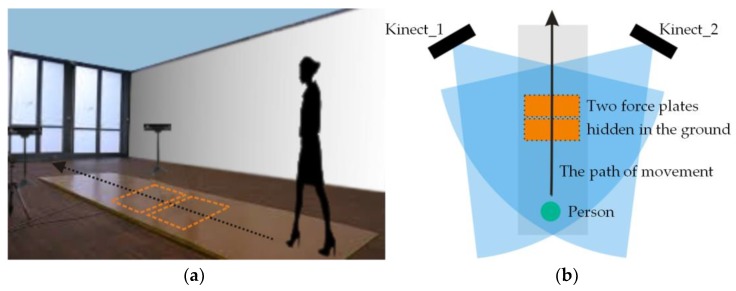
Diagram of human gait measurement: (**a**) a perspective view; (**b**) a view from above.

The study was carried out at the Bialystok University of Technology on a group of 99 women aged 21.48 ± 1.17 with a body weight of 61.90 ± 11.07 kg and a body height of 166.41 ± 5.74 cm. All participants were informed about the aim and course of the experiment and signed a consent form. During the research the women walked through a measuring path with two hidden force plates manufactured by the Kistler Company. The participants were not informed about the presence or about the location of the plates nor about having to step on one. In the event when the test subject did not tread on the platform or stepped on its edge the measurement was repeated with a slight adjustment of the starting point of the test. Additionally, two Microsoft Kinect v2 devices were used to record the person’s body height. The devices were placed more or less symmetrically in relation to the walking path of the test subject who moved toward them. The two devices were not concealed in any way (Figure 7).

Each of the analyzed subjects walked in their own footwear: sports shoes and high heeled shoes with the heel height specified to be from 8 to 10 cm. Testing with both types of footwear was conducted on the same day. During the experiment, after every 10 gait strides with a single person there was a short, 1–2 min, break to avoid the subject becoming tired. 14 to 20 gait cycles were carried out for each type of footwear with every participant. In total 3402 strides were recorded (1874 cycles for sport shoes and 1528 cycles for high heels).

Additionally, to ensure the robustness of the proposed method, a secondary study was performed on a group of 6 women. The selected ladies were tested after a period ranging from 3 to 12 months from the date of the first test. During the second test the women, for the most part (5 of the 6), used the same footwear as during the first series of tests. In the first series of tests 201 strides were recorded for this sub-group and as the results of the secondary testing 203 strides were recorded. In respect to this sub-group the selected footwear recognition classifier (see Figure 6) was trained on data describing the gait of all 93 people taking part in the experiment.

Since the set of people who participated in secondary test is relatively small obtained results of recognition may not be representative. Hence these results will be compared in relation to the sub-group of the selected 6 women (meaning the recognition results on the basis of the 1st test vs. the 2nd test) and discussed separately.

## 4. Results

Testing of classifiers which made the identification of footwear was conducted with the help of the WEKA software and the cumulative results obtained for test runs have been presented in Table 2. Parameters characterizing gait in high heels was selected as the relevant class, and sensitivity and specificity were calculated using the following formulas:

(6) $$\mathrm{Sensitivity}=\frac{TP}{\left(TP+FN\right)}\cdot 100\%$$

(7) $$\mathrm{Specificity}=\frac{TN}{\left(TN+FP\right)}\cdot 100\%$$

where *TP*—the number of true positives (correctly recognized strides of people walking in high heels); *FN*—the number of false negatives (gait strides of people walking in high heels which have been recognized as gait strides of people walking in sport shoes); *TN*—the number of true negatives (correctly recognized gait strides of people walking sport shoes); *FP*—the number of false positives (gait strides of people walking in sport shoes which have been recognized as gait strides of people walking in high heels).

The best results were seen with the SVM classifier while the worst were seen with the Naive Bayes. A high CCR value was also obtained using the feedforward neural network. However, a higher standard deviation caused the authors to utilize SVM in further work. A very high specificity value was reached by the kNN classifier (*k* = 3, city blocks) but its lowest sensitivity value caused it to be excluded from further work. Slightly higher specificity than sensitivity values for all classifiers were an expected result and stemmed from the fact that walking in high-heeled footwear is characterized by a greater variability within classes than walking in sport shoes. It is also worth mentioning that CCR of most classifiers oscillated around 95–96%.

The following scenarios were considered within the framework of this study:

(a) Data contained only the gait of people wearing sport shoes and used solely measurements from force plates;
(b) Data within the training set contained only gait in sport shoes while the testing set included all other data but the classification was done solely on the basis of GRF (without Microsoft Kinect v2 measurements);
(c) Same as in point (a) but with identification of footwear type and body height of the person being identified;
(d) Same as in point (b) but with identification of footwear type and body height of the person being identified (as described in Materials and Methods);
(e) Same as in point (d) but with the assumption that the identification of footwear will be at 100% accuracy.

In order to enable the comparison of gathered results with the outcomes of other authors randomly selected results for varying number of people from 10 to 90 in increments of 10 (10, 20, 30, …, 90) as well as for all people participating in the experiment were presented. In order to reduce the impact of randomness on results the tests were repeated 10 times for every group of people. On the basis of preliminary studies the number of considered nearest neighbors *k* equaled to 5. Number of gait cycles in the testing set varied and depended on the number of people considered in a particular test.

Assumptions defined in scenario (d) were applied in respect to the sub-group of women with whom secondary testing was performed. In this case, the training set was data from all 99 people, drawn in accordance with the methodology described in subsection 3.5. The testing set was data from the second series of the experiment.

**Figure 8:**
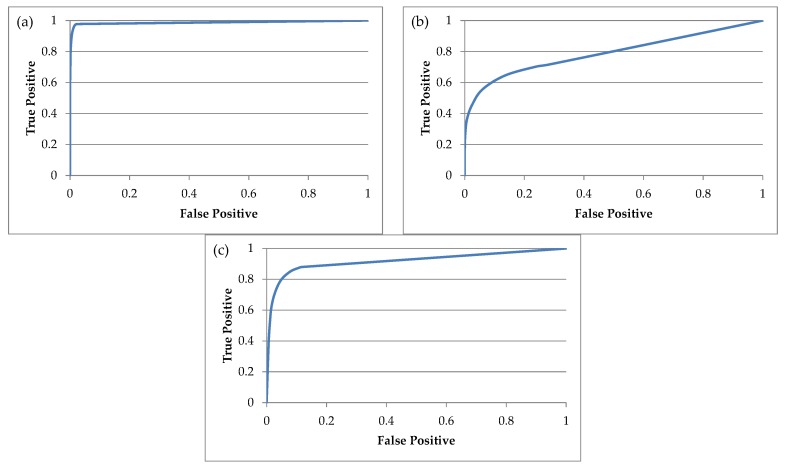
The ROC curves in case of 99 subjects for: (**a**) scenario (a) AUC = 0.987; (**b**) scenario (b) AUC = 0.789; (**c**) scenario (d) AUC = 0.921. AUC = Area Under Curve.

The results presented below assume the acceptance of the most liberal strategy where *Th* = 0. Data in tables (Table 3 and Table 4) a presents Correct Classification Rates, False Rejected Rates (FRR) and False Accepted Rates (FAR). Figure 8 consists of the ROC curve for scenarios (a), (b) and (d).

Data from the table above should be treated as a reference in relation to the proposed method. Results achieved in scenario (a) confirm that in cases where the training set as well as the testing set contained measurements of gait in the same type of footwear then the accuracy of classification is very high and only single cycles are assigned to other people. It should be added that in the majority of bad classifications the weighted total has a value which is significantly lower than in cases of correct classifications. Therefore, in establishing the value of the threshold *Th* it is very easy to reduce the error value of FAR with the obvious increase in the error of FRR. In turn, data from scenario (b) demonstrates that the usefulness of gait biometrics with such a drastic change of footwear type is small even with relatively small data sets.

The goal of scenario (c) was to show the impact of the effect of the classifier recognizing footwear which the person being tested was wearing. Obviously, since this classifier does not have 100% correct classifications the results here are less accurate than those from scenario (a). They are also quite surprising since increasing the number of people within a group has practically no impact on the final results. The differences between particular amounts of people result from the random character of selecting these people to the given group. Additionally, some badly classified standards find their way into the training set (gallery) and do not influence the results negatively. It must also be added that our observations are confirmed by the spread of CCR between individual samples for particularly small sets.

The effectiveness of the proposed method is most aptly demonstrated by the values obtained with scenario (d). The larger the group of participants the greater the difference between values of scenario (b) and (d). The relatively small CCR value for a group of 10 people may cause concern but similar to the other scenarios it is the result of the random selection of people for the group (in individual samples CCR varied from 90.84 to 98.48%). Scenario (e) presented results in cases where the classifier identifying footwear type worn by the test subject worked with 100% accuracy. It shows the potential of the presented method and suggests the best results which could be obtained on the basis of measurements gathered in this study and without changing applied base classifiers.

In relation to the sub-group of 6 women who took part both in the first series of tests as well as in secondary testing the footwear recognition classifier correctly identified 95.02% and 97.04% of footwear in recorded walking cycles. These values are at similar levels to those presented in Table 2. The recognition accuracy of people from this group after the application of the procedure described in scenario (d) has been presented in Table 5.

The resulting values show that there was only a slight decrease in the accuracy of people recognition on the basis of gait data recorded a few months later. It is smaller from the expected and natural for behavioral biometrics. It is worth pointing out that the higher than average level of footwear recognition worn by the person subjected to the tests plays a certain positive role in all of this. Because this phenomenon may be incidental then, in general, a CCR below 91% for a given group of people should be expected. Generally, it must be said that the proposed biometric system turned out to be relatively resistant to the passing of time.

## 5. Discussion

The obtained results are already very good. Results shown in scenario (b) are noticeably better in comparison to [46]. It is the effect of reducing the number of base classifiers through excluding classifiers operating on data from the first and last gait sub-phases registered through the platform (loading response and pre swing). GRF values in those phases are relatively low. This, in many cases, causes the intra-individual variability to be greater than inter-individual variability which, in turn, leads to low values of CCR in base classifiers responsible for recognizing people on the basis of time series of those phases and, in consequence, impacts negatively the recognition accuracy of the entire team of classifiers.

Results gained through the use of the proposed method (scenario (d)) are considerably better than those reported in work of other authors dealing with similar topics [51,64,65]. They are, in fact, superior also because, for example, Connor tested only men and gait in men’s formal footwear does not significantly vary from walking in sport shoes which, as has been shown in [51], has a smaller impact on classification results. In turn, in the work of Connie et al. the test set used data describing the gait of both women as well as men, however, lack of information about the percentage of women in the study group and the large number of participants (125) makes comparison of results difficult. Nevertheless, it does seem that the presented method would achieve better results with a similar group of people. It is also worth mentioning that in two of these works different measuring systems were utilized: motion capture system [51] and video cameras [65]. Similar signals were considered in Connor’s work but were additionally augmented with spatial features and signals derived from high-resolution sensing floor tile.

Unfortunately the method being discussed also possesses limitations. Its weaknesses undoubtedly include the tightly defined heel height. In real situations and with the number of people being considered it would be highly probable that there would be people who would wear shoes with lower heels. The direct application of the proposed method and the reduction of the body height of such a person could have caused not being able to properly identify her. Such cases would require the algorithm to be altered either through adding another type of footwear as a potential class recognized in the first stage of the method or through replacing the classifier with an approximator generating on its output a particular value by which the person’s body height would need to be modified.

## 6. Conclusions

Within this article we have presented the workings of a biometric system dependent on the type of footwear worn by women—sport shoes or high heels. It has been shown that in cases where gait in high heels is not included in the learning set of the ensemble classifiers then the accuracy of the biometric system is lower even with a relatively small study group than the precision of the same system with a large group of women walking only in sport shoes. However, the obtained results are very good and demonstrated a significant improvement in the quality of a biometric system in comparison to reports currently available in literature. The robustness of the proposed method is especially worthy of attention.

Further work in this area can be carried out in two directions. First, the database needs to be enhanced with data presenting the gait of men and women in several different types of footwear. Secondly it is necessary to seek feature extraction methods or classifications which will improve the results presented within this study.

## Figures and Tables

**Table 1:** Technical specification of the Kinect v2 sensor.

Feature	Kinect v2
Color camera	1920 × 1080 × 16 bit per pixel 16:9 YUY2; 30 Hz (15 Hz in low light, HD)
Depth camera	512 × 424 × 16 bits per pixel 16-bit ToF depth sensor IR can now be used at the same time as colour
Working range	Only one configuration: 0.5 m to 8 m; Quality degrades after 4.5 m
Angular field of view	60° vertical; 70° horizontal
Skeletal joints	25 joints tracked; 5 more than the Kinect for Windows V1: Neck, left and right Thumbs and Hand Tips
Maximum skeletal tracking	6 with joints (renamed to Bodies)
Method of depth measurement	Time of Flight

**Table 2:** The average value of Correct Classification Rate, Sensitivity and Specificity ± SD for different types of classifiers.

Type of Classifier	CCR	Sensitivity	Specificity
kNN	95.27 ± 4.11	90.98 ± 7.18	98.71 ± 2.09
Naïve Bayes	93.81 ± 4.76	91.85 ± 6.46	95.70 ± 5.68
**SVM**	**96.43 ± 3.09**	**95.80 ± 5.51**	**96.82 ± 4.80**
ANN	96.13 ± 4.14	96.07 ± 5.97	96.09 ± 3.85
Random Forest	95.77 ± 3.62	94.10 ± 6.15	97.26 ± 3.19
Deep ANN	93.94 ± 6.13	95.87 ± 5.20	91.97 ± 11.82

**Table 3:** Correct Classification Rate, False Rejected Rate and False Accepted Rate for the reference scenarios: (a) and (b).

No. of Sub.	Scenario (a)			Scenario (b)		
	CCR	FRR	FAR	CCR	FRR	FAR
10	99.09	0	0.91	92.70	0	7.90
20	99.21	0.04	0.04	86.81	0.27	12.92
30	98.60	0	0	83.92	0.23	15.86
40	98.53	0	0	81.49	0.39	18.12
50	98.45	0.06	0.06	77.27	0.56	22.17
60	97.86	0.08	0.08	75.48	0.48	24.04
70	98.15	0.04	0.04	74.98	0.65	24.37
80	97.96	0.06	0.06	73.53	0.69	25.78
90	97.73	0.11	0.11	72.20	0.75	27.05
99	97.74	0.06	0.06	71.62	0.73	27.65

**Table 4:** Correct Classification Rate, False Rejected Rate and False Accepted Rate for scenarios: (c), (d) and (e).

No. of Sub.	Scenario (c)			Scenario (d)			Scenario (e)		
	CCR	FRR	FAR	CCR	FRR	FAR	CCR	FRR	FAR
10	97.93	0.58	1.49	95.38	0.82	3.80	97.48	0	2.52
20	95.82	0.31	3.87	94.63	0.59	4.78	98.13	0	1.87
30	97.29	0.03	2.67	92.76	1.16	6.08	96.28	0.04	3.68
40	97.06	0.02	2.91	92.72	0.71	6.56	95.77	0.11	4.12
50	96.68	0.06	3.26	90.93	0.70	8.37	95.91	0.05	4.38
60	96.22	0.03	3.75	90.62	0.56	8.83	93.85	0.06	6.09
70	96.57	0.03	3.40	89.83	0.66	9.51	93.29	0.10	6.61
80	96.71	0.05	3.24	89.14	0.60	10.26	93.19	0.13	6.68
90	96.64	0.02	3.34	88.70	0.58	10.71	92.27	0.16	7.57
99	96.47	0.01	3.52	88.27	0.59	11.14	91.42	0.17	8.41

**Table 5:** Correct Classification Rate, False Rejected Rate and False Accepted Rate for subgroup of six women for the first and second series of tests.

Experiment	CCR	FRR	FAR
First test	92.06	0.06	7.88
Second test	91.53	0.07	8.40
